# Value Assessment of Ecosystem Services in Nature Reserves in Ningxia, China: A Response to Ecological Restoration

**DOI:** 10.1371/journal.pone.0089174

**Published:** 2014-02-19

**Authors:** Yan Wang, Jixi Gao, Jinsheng Wang, Jie Qiu

**Affiliations:** 1 College of Water Science, Beijing Normal University, Beijing, China; 2 Nanjing Institute of Environmental Science, Ministry of Environmental Protection, Nanjing, China; DOE Pacific Northwest National Laboratory, United States of America

## Abstract

Changes in land use can cause significant changes in the ecosystem structure and process variation of ecosystem services. This study presents a detailed spatial, quantitative assessment of the variation in the value of ecosystem services based on land use change in national nature reserves of the Ningxia autonomous region in China. We used areas of land use types calculated from the remote sensing data and the adjusted value coefficients to assess the value of ecosystem services for the years 2000, 2005, and 2010, analyzing the fluctuations in the valuation of ecosystem services in response to land use change. With increases in the areas of forest land and water bodies, the value of ecosystem services increased from 182.3×10^7^ to 223.8×10^7^ US$ during 2000–2010. Grassland and forest land accounted for 90% of this increase. The values of all ecosystem services increased during this period, especially the value of ecosystem services for biodiversity protection and soil formation and protection. Ecological restoration in the reserves had a positive effect on the value of ecosystem services during 2000–2010.

## Introduction

Ecosystem services (ESs) refer to the benefits people obtain from ecosystems or to aspects of ecosystems used (actively and passively) to produce human well-being [1], [2]. ESs emphasize not only provisioning services (marketable goods), but also supporting (e.g. nutrient cycling), regulating (e.g. soil and water conservation), and cultural services (e.g. aesthetic values). As the Millennium Ecosystem Assessment [1], [3] reminded us, our lives, much less our societies, economies, or well-being, depend on ESs [4]. Ecosystem service values (ESVs) are the values of the contributions of ESs to the sustainability of human well-being [5]. The growing body of literature on ESV includes studies on the value generated by ecosystems [6], [7], changes in ESVs in response to changes in land use [8], [9], climate change [10], [11], approaches and models for assessing ESV [12], [13], and other factors [14], [15].

Ecosystems have been substantially exploited, degraded, and destroyed in the last century as a consequence of the global increase in economic and societal prosperity [1]. Humans have changed ecosystems more rapidly and extensively in the last 50 years than in any comparable period of human history; some 60% of the ESs studied have been degraded during this period [1], [16]. The concept of ESs has become a central issue in conservation planning and the assessment of environmental impacts for preventing further abatement of the quality of ecosystems [17], [18]. Many case studies on ESs have been performed, but too few have paid enough attention to long-term fluctuations of ESs, even though long-term study is necessary for detecting the response of ESs to land use change, climate change, or other factors. Monitoring the fluctuations in ESVs would benefit the management and maintenance of ecosystem sustainability, e.g. the identification and measurement of variations in ESs as a consequence of land use changes appears to be an adequate means of evaluating the environmental costs and benefits of decisions affecting the planning of land use [19].

The autonomous region of Ningxia is a typical region of western China, and harsh natural conditions and the over-exploitation of resources have deteriorated the already fragile ecological environment in the past few decades. The Chinese government has recently taken measures to improve the ecological environment, such as the conversion of cropland to forest, prohibition of enclosures and grazing, and sand prevention and control. Estimating the effects of these measures is thus essential for restoring ecologically fragile regions. The effects of the restoration efforts during 2000–2010 in western China, the first decade of such efforts, have generated concern. We have assessed the variation of ESVs based on land use change in the national nature reserves in Ningxia to determine the effects of ecological restoration.

The unit prices of ESVs in global biosphere ecosystems were estimated by Costanza et al. [20]. ESVs are now commonly estimated by integrating the use of the Geographic Information System (GIS) and remote sensing (RS) [21], [22], [23]. The estimates of Costanza et al. [20] have been criticized because they overestimate ESVs for wetland and underestimate ESVs for farmland [24], [25]. A survey of Chinese ecologists led to an improved approach suitable for the situation in China [26]. This approach includes merging some ES functions, as suggested by Costanza et al. [20], [27], and extracting the equivalent weighting factors for ESs per hectare for terrestrial ecosystems [28]. Much of the research on ESVs based on the equivalent weighting factors and land use data suggest that land use type can be a proxy for ESs by matching the types to the equivalent biomes, which is convenient and simple for ESVs of large areas [29], [30], [31].

Based on a decade of RS data combined with equivalent weighting factors and the use of ArcGIS, a package of programs for working with GIS data, this study assessed the ESVs of national nature reserves in Ningxia for the years 2000, 2005, and 2010. Our objectives were to: (1) analyze the changes in land use in the reserves during the past 10 years, (2) assess the variation in ESVs in the reserves during this period, and (3) discuss the effects of ESVs on ecological restoration.

## Materials and Methods

### Study Site

**Figure 1:**
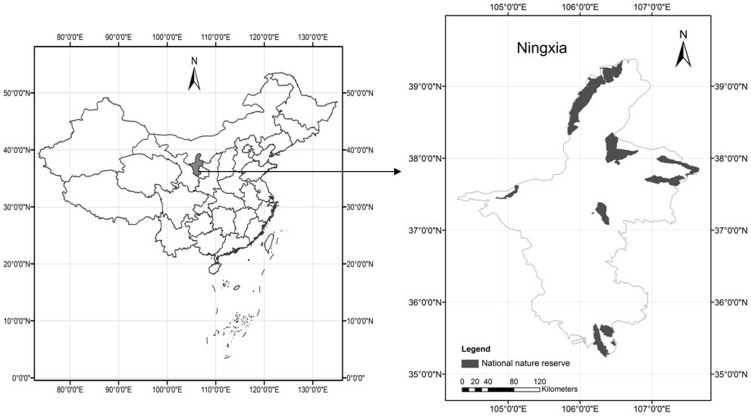
Location of the study site.

The study area included six national nature reserves in Ningxia, which is located in western China at 35°25′N–39°22′N and 104°49′E–107°40′E (Figure 1). The area is in a temperate arid/semi-arid region that has a continental monsoon climate, four distinct seasons, and abundant sunshine. The area is geographically diverse with annual average temperatures of −0.9 to 9.6°C and an annual precipitation of 186–800 mm. The multi-type nature reserves in the study site are classified in China as forest, wetland, and desert ecosystems and contain approximately 1000 species of plants and vertebrates in the six nature reserves. The abundant variety of natural resources provides a variety of ESs.

### Data Collection and Land Use Classification

An integrated approach utilizing a Geographic Information System (GIS) and Remote Sensing (RS) was used to extract a data set for the changes in land use. The data set was extracted from GIS and RS data from Landsat Thematic Mapper imagery for 2000 and 2005 and from environmental satellite data (http://www.secmep.cn/secPortal/portal/index.faces) for 2010. To maintain consistency of classification with the remote sensing data, all the remote sensing images with good image quality in July were selected for analysis. After converting the data to the unified coordinate system and projection, we used the Krasovsky ellipsoid and the Transverse Mercator projection of ENVI 4.8 to perform RS image radiation correction and geometry correction, respectively.

After completing the pretreatments, including image mosaics, grooming, and data fusion, we used ArcGIS10.0 to consolidate and analyze the land use data with a background of raster images. The maximum likelihood classifier of the supervised classification method was used for classification with ENVI 4.8. According to the confusion matrix for classification accuracy, qualitative errors of precision for deciphering the remote sensing data for different years were controlled at the 90% level. Comparing the results of the interpretation with those of the field survey of typical points, the total classification accuracies were all higher than 90%, and the total Kappa coefficients were all greater than 0.8, which were higher than the minimum acceptable (0.7). The accuracy could thus meet the monitoring accuracy of the demands for land use change. Our data set included seven classified land use types listed in the resource and environmental database established by the Chinese Academy of Sciences (Table 1). ArcGIS 10.0 and SPSS 19.0 were used for the statistical analysis.

**Table 1:** Definitions of land use type in the national nature reserves in Ningxia.

Type	Definition
Forest land	Arbor, bush forest, broad-leaved forest, coniferous forest, and mixed forest
Grassland	Meadow and steppe
Farmland	Dry land, irrigable land, and crop fields
Wetland	Herbaceous swamp and thicket swamp
Water body	Rivers, ponds, reservoirs, and lakes
Construction land	Land used for industry, residences, and transportation
Unused land	Bare soil, bare rock, and saline-alkali soil

**Figure 2:**
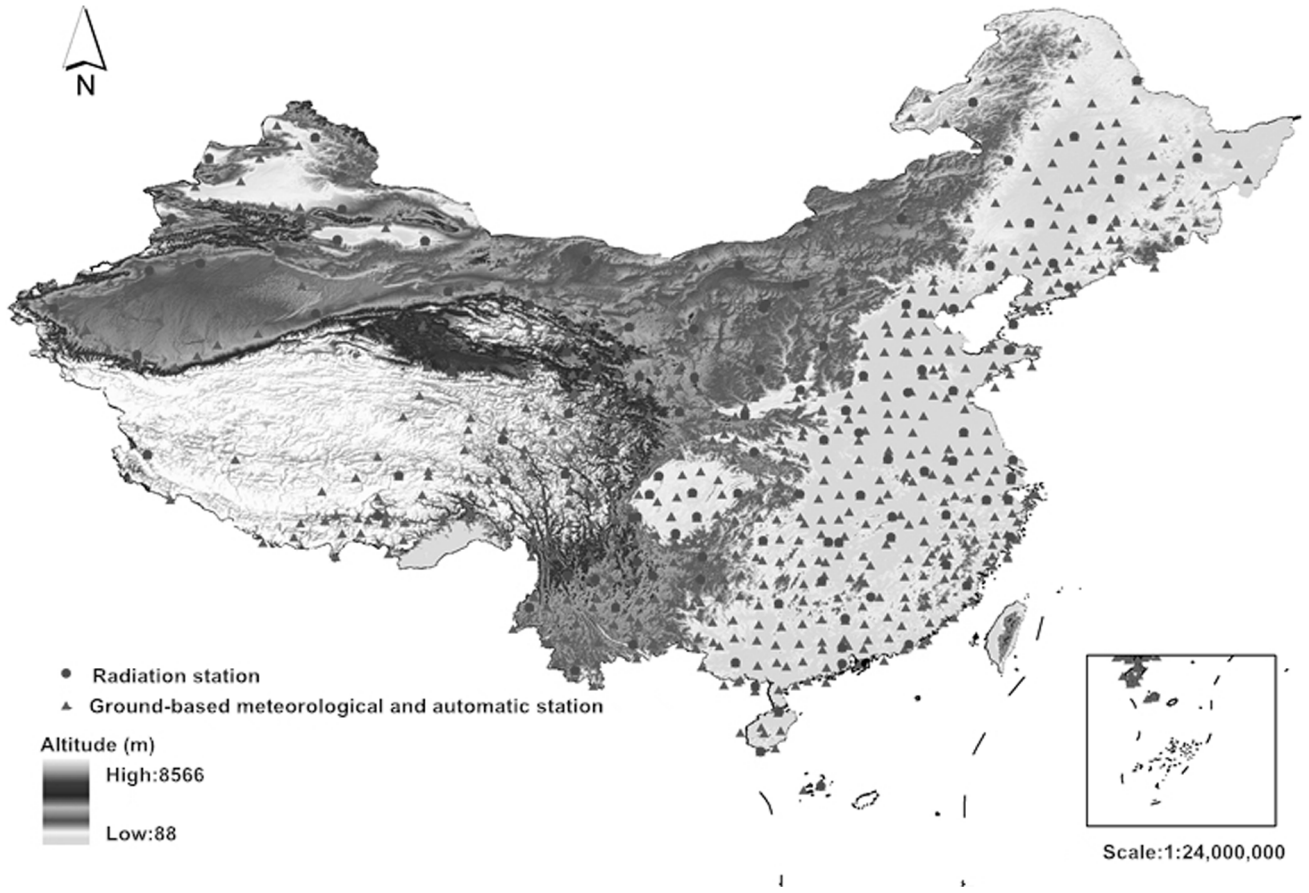
Distribution of weather stations.

We obtained data sets for the normalized difference vegetation index (NDVI) from the Goddard Space Flight Center (NASA) (http://ladsweb.nascom.nasa.gov/data/search.html). We obtained climatic data sets, including the monthly records from 122 radiation stations and 756 ground-based meteorological and automatic stations, from the web of China meteorological data sharing service (Figure 2, http://cdc.cma.gov.cn/index.jsp). Data for vegetation types were obtained from GLC2000 China regional land cover data (http://solargis.info/doc/32).

### Land Use Dynamics

ESVs were determined by the value coefficients and areas of land use types. The changes of areas directly caused the variation in ESVs. The areas were statistical calculations of the areas of various land use types from the remote sensing data, so the remote sensing data were the basis of the calculated ESV data and had a direct relation with the ESV calculation. Images of the national nature reserves in Ningxia from 2000, 2005, and 2010 were used to estimate the land use changes in the past decade. The Map Algebra function of ArcGIS10.0 was used to calculate the figures of land use type for 2000, 2005, and 2010 and the dynamics of land use. The rate (R) of change of land use was calculated as:
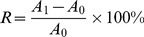
(1)where *A_0_* and *A_1_* represent the initial and final areas of a given land use, respectively.

### ESV Assessment

Costanza's [20] theory and the survey of 500 Chinese ecologists [26] suggested that the equivalent value per unit area (Table 2) was practicable in China, and it has been widely used to assess ES [28], [32], [33]. The ESV of one equivalent weight factor was calculated as [34]:
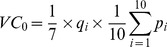
(2)where *VC_0_* is the economic value of one equivalent weight factor (Yuan·ha^−1^·yr^−1^)(1 Yuan = 0.16 US$), *q_i_* is the average grain price nationwide (Yuan·kg^−1^), *p_i_* is the yield of per unit area of crops in year *i* (kg·ha^−1^·yr^−1^), and *i* is the year.

**Table 2:** Equivalent value per unit area of ecosystem services in China [26].

	Forest land	Grassland	Farmland	Wetland	Water body	Unused land
Gas regulation	4.32	1.50	0.72	2.41	0.51	0.06
Climate regulation	4.07	1.56	0.97	13.55	2.06	0.13
Water supply	4.09	1.52	0.77	13.44	18.77	0.07
Soil formation and protection	4.02	2.24	1.47	1.99	0.41	0.17
Waste treatment	1.72	1.32	1.39	14.40	14.85	0.26
Biodiversity protection	4.51	1.87	1.02	3.69	3.43	0.40
Food production	0.33	0.43	1.00	0.36	0.53	0.02
Raw material	2.98	0.36	0.39	0.24	0.35	0.04
Recreation and culture	2.08	0.87	0.17	4.69	4.44	0.24
Total	28.12	11.67	7.90	54.77	45.35	1.39

Species resources, especially for rare species, are much more abundant inside than outside nature reserves, so adjusting the equivalent value per unit area of biodiversity protection is essential. A database of 3337 rare and endangered species in China was established from lists of national key protected wildlife species, the CITES (Convention on International Trade in Endangered Species) appendix, IUCN (the International Union for Conservation of Nature) endangered species level 3.1, a list of China's endemic species, and the IUCN Red List of Threatened Species. Based on information of species protection in 861 nature reserves in China, the distributions of 2157 rare and endangered species were determined for the nature reserves. Information about protection in national nature reserves accounted for 96.2% of the objects and 99.7% of the area, indicating that the calculated value per unit area was better representative of the important species. We then used the density of important species as a parameter for correction, calculated as:
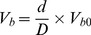
(3)where *V_b_* is the equivalent value per unit area of biodiversity protection in nature reserves, *d* is the density of important species in nature reserves (species·ha^−1^), *D* is the average distribution density of important species on a national scale (species·ha^−1^), and *V_b0_* is the equivalent value per unit area of biodiversity protection in China.

The equivalent value per unit area of ES was based on the national average, so we calibrated for regional differences when assessing the ESV of a local area. Because ESV is closely related to ecosystem productivity, we used a correction factor based on ecosystem productivity to adjust the calculation of ESV. The regional correction factor reflected the difference in net primary production (NPP) caused by the variations in climate between local areas and the country as a whole. The ESV of one unit area for a year was calculated as:
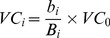
(4)where *b_i_* and *B_i_* are the average NPPs of ecosystems in the study areas and the country in year *I*, respectively, and *i* is 2000, 2005, or 2010.

The CASA (Carnegie-Ames-Stanford Approach) model is based on the principle that plant productivity is correlated with the amount of photosynthetically active radiation absorbed or intercepted by green foliage [35], [36]. Transformation to regional scales is easily achieved by the model, which is valuable for estimating the annual dynamic change in NPP on regional scales. RS data can provide information on many parameters of the vegetation. We obtained the parameter *FPAR* (see below) required for calculating NPP from time-series data for NDVI from the MODIS spectroradiometer aboard the EOS satellites. The climatic data, including total solar radiation, average temperature, and duration of sunshine, were obtained from 122 radiation stations and 756 ground-based meteorological and automatic stations in China. The equations we used were:
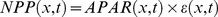
(5)

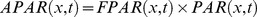
(6)

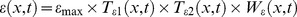
(7)where *APAR* is photosynthetically active radiation (mJ·m^−2^); *ε* is the actual light use efficiency of vegetation (g·mJ^−1^); *x* and *t* refer to location and time, respectively; *PAR* is the total incident photosynthetically active radiation (mJ·m^−2^); *FPAR* is the fraction of *PAR* absorbed by the vegetation canopy; *ε_max_* is the maximum light use efficiency under ideal conditions (g·mJ^−1^); *T_ε1_* and *T_ε2_* refer to stress effects of low and high temperatures on the use efficiency of light energy, respectively; and *W_ε_* is the water stress influence coefficient, which represents the influence of moisture conditions.

The ESVs of each land use type and service function and the total ESV were then calculated as:
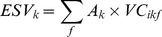
(8)

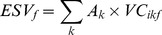
(9)

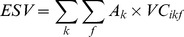
(10)where *ESV_k_*, *ESV_f_*, and *ESV* refer to the ESVs of land use type *k*, service function *f*, and the ecosystem (Yuan·ha^−1^), respectively; *A_k_* is the area of land use type *k* (ha); and *VC_ikf_* is the value coefficient for land use type *k* with ES function type *f* (Yuan·ha^−1^) in year *i*.

The contribution rate used to assess the effect of ESV variation on land use change was calculated as [20]:
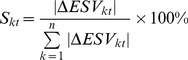
(11)where *S_kt_* is the proportion of the absolute value of ESV variation of land use type *k* in period *t* to the total amount of ESV variation of land use type *k* in period *t*.

### Sensitivity Analysis of ESV

The coefficient of sensitivity (CS) validates the land use types representative of ecosystem type and certainties in the value coefficients [8], [37], [38]. CS takes the response of ESV to the ecological value of changes in unit price as a measure of the degree of sensitivity of ESV to a coefficient. CS was calculated as:
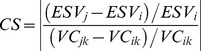
(12)where *ESV_i_* and *ESV_j_* are the initial and adjusted total ESVs, respectively, and *VC_ik_* and *VC_jk_* are the initial and adjusted value coefficients, respectively. ESV is considered to be unaffected by the coefficient, and the results will be reliable when CS<1, and ESV is considered elastic relative to the coefficient when CS>1. Larger values of CS will define *VC*s more accurately. Regardless of how the value coefficients change, the sensitivity of ESV to changes in the value coefficients must be kept low to ensure the reliability of our results. To verify CS, a 50% adjustment in the value coefficients was made to estimate the percent changes in the calculated total ESV and the CSs.

## Results

### Changes of Land Use

**Figure 3:**
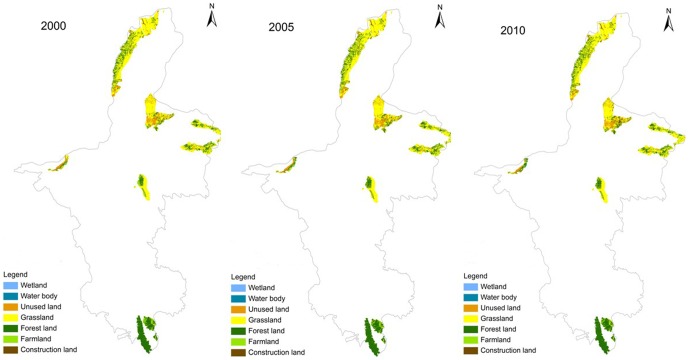
Distribution of land use types during 2000–2010.

**Figure 4:**
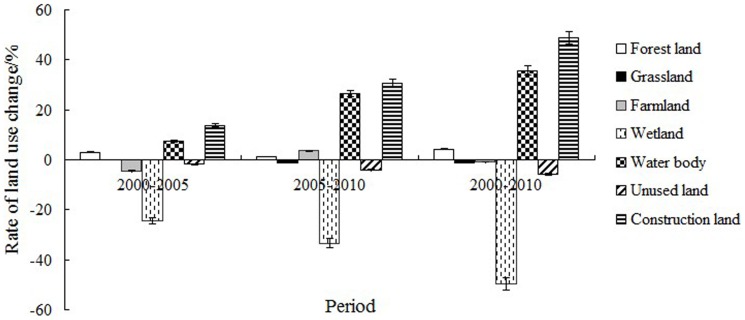
Dynamic rates of each land use type during 2000–2010.

Table 3 and Figure 3 show the land use changes in the national nature reserves in Ningxia during 2000–2010. The area of grassland was an important factor in the reserves, accounting for approximately 60% of the total land area. The areas of wetland and unused land decreased during this period by 49.7% and 5.7%, respectively (Figure 4). The area of grassland increased during 2000–2005, decreased during 2005–2010, and had decreased by 1.1% by the end of the decade. The area of farmland decreased during 2000–2005, increased during 2005–2010, and had eventually decreased by 0.58% by the end of the decade. The areas of forest land, water bodies, and construction land continuously increased throughout the decade, by 4.3, 35.8, and 48.8%, respectively. The amount of wetland in the reserves declined more sharply than did the other land use types. The amount of construction land rose sharply and had the highest rate of increase due to the increasing encroachment of human activities.

**Table 3:** Areas of land use types in the national nature reserves in Ningxia in 2000, 2005, and 2010.

Land use type	2000		2005		2010	
	Area(ha)	Percentage(%)	Area(ha)	Percentage(%)	Area(ha)	Percentage(%)
Forest land	59809.5	14.01	61652.5	14.44	62374.9	14.61
Grassland	254377.2	59.58	254469.9	59.61	251606.6	58.94
Farmland	43310.2	10.14	41412.9	9.70	42941.3	10.06
Wetland	65.9	0.02	49.8	0.01	33.2	0.01
Water body	1989.5	0.47	2137.1	0.50	2701.3	0.63
Unused land	60476.3	14.17	59360.9	13.90	57013.7	13.35
Construction land	6887.4	1.61	7833.0	1.83	10245.0	2.40
Total	426916	100.00	426916	100.00	426916	100.00

### Changes of ESV

**Figure 5:**
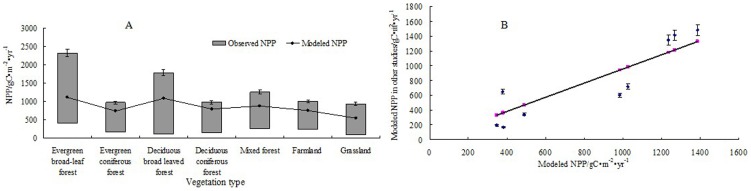
A. Relationship between modeled NPP and observed NPP. B. Relationship between modeled NPP and other evaluations.

**Figure 6:**
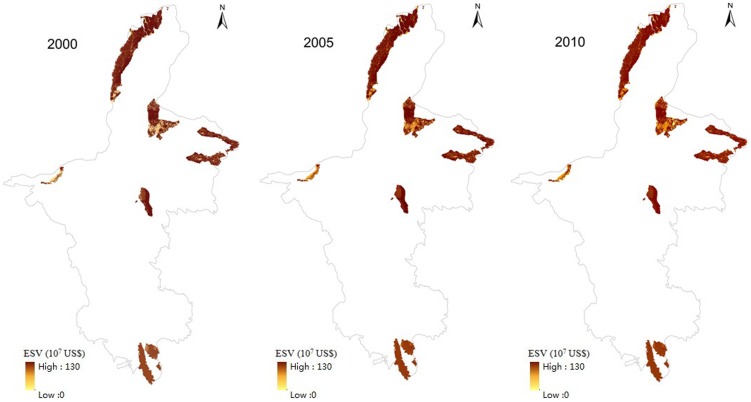
Ecosystem services values of land use types during 2000–2010.

The range of annual mean NPP was determined based on the observed NPP data of different vegetation types, including evergreen broad-leaved forest, evergreen coniferous forest, deciduous broad-leaved forest, deciduous coniferous forest, mixed forest, grassland and farmland [39], [40], [41]. The modeled NPP values were all within the range of observed values (Figure 5 A), indicating that the modeled result was consistent with the actual NPP. The modeled NPP values between 2000 and 2010 from other studies were compared with this study through correlation analysis [41], [42]. The correlation coefficient was *R* ^2^ = 0.81 (Figure 5 B), showing that the modeled NPP in this study was in agreement with other studies. Through the above validation, we concluded that the NPP calculation by CASA in this study was reliable. The NPP values of terrestrial ecosystems in China calculated with the CASA model were 689, 711, and 692 gC·m^−2^·yr^−1^ in 2000, 2005, and 2010, respectively. The NPP values of terrestrial ecosystems in Ningxia were 384, 441, and 468 gC·m^−2^·yr^−1^ in the three years, respectively. The ESVs of each land use type in 2000, 2005, and 2010 are shown in Table 4 and Figure 6 (calculated with Eqs. 1–4, 8–10). The total ESV of the reserves in Ningxia increased throughout 2000–2010 by 22.7%. Total ESV increased at a higher percentage during 2000–2005 than during 2005–2010, by 12.3 and 9.2%, respectively.

**Table 4:** Ecosystem service values (ESVs) and their variation for each land use type in the national nature reserves in Ningxia in 2000, 2005, and 2010.

Land use type	2000		2005		2010	
	ESV (10^7^ US$)	Percentage(%)	ESV (10^7^ US$)	Percentage(%)	ESV (10^7^ US$)	Percentage(%)
Forest land	59.57	32.67	68.42	33.40	75.43	33.70
Grassland	105.11	57.64	117.16	57.20	126.22	56.40
Farmland	11.13	6.11	11.86	5.79	13.40	5.99
Wetland	0.06	0.03	0.08	0.04	0.10	0.04
Water body	2.49	1.37	2.98	1.46	4.11	1.84
Unused land	3.96	2.17	4.34	2.12	4.54	2.03
Construction land	0	0	0	0	0	0
Total	182.3	100.00	204.8	100.00	223.8	100.00

**Figure 7:**
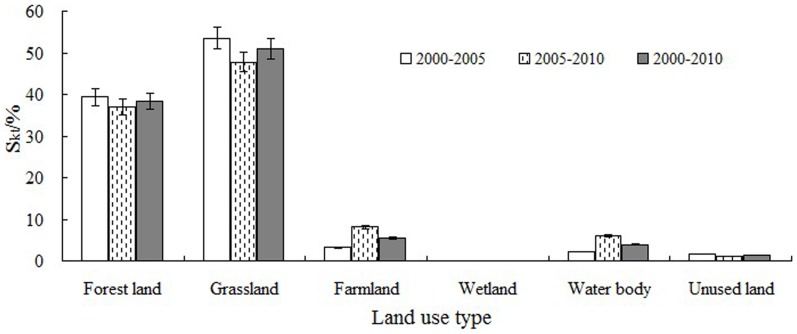
Contribution rate of each land use type during 2000–2010.

The ESV of grassland, with such a large area, contributed most to the total ESV, accounting for approximately 57%. The ESV of forest land, accounting for approximately 33% of the total, continually increased during the decade. The ESVs of forest land and grassland thus constituted a substantial portion of the total ESV. The contribution rates used to assess the effect of ESV variation on land use change are shown in Figure 7. Grassland contributed most in nearly every period, indicating that changes in the area of grassland had the strongest influence on the variation in ESV. The areas and influences of forest land and farmland were also relatively large. The area of water bodies was much lower than the area of unused land, but the ESV of water bodies was nearly that of unused land, with a high value coefficient. The contribution rates of water bodies were higher than those of unused land in every period.

**Figure 8:**
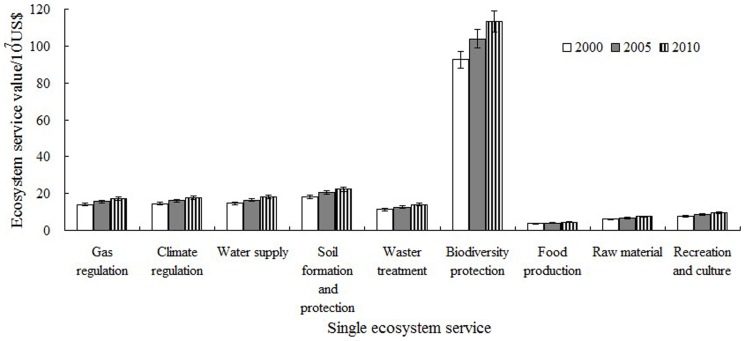
Values of ecosystem services during 2000–2010.

The ESVs of the ESs in the reserves are shown in Figure 8. The ESVs of each ES increased throughout 2000–2010. Biodiversity protection contributed most to the total ESV, accounting for more than 50% of the total value in the decade, indicating the effect of nature reserves on biodiversity protection. The value of food production was the lowest, accounting for less than 2% of the total, and demonstrated the importance of the policies to protect nature reserves from commercial exploitation. The value of biodiversity protection increased most during the period. The areas of forest land and water bodies increased sharply with high value coefficients, showing good effects on habitat supply. The ESV of soil formation and protection increased during this period, indicating that conditions of soil desertification in the reserves in Ningxia had improved. The Wilcoxon Signed Ranks Test of SPSS 19.0 showed that the ESVs of various land use types increased significantly in different periods, and the ESVs of each ecosystem service increased quite remarkably in different periods (Table 5). These increases indicated that the ESVs of the study area increased significantly during 2000–2010.

**Table 5:** Difference analysis of ecosystem service values (ESVs) in the national nature reserves in Ningxia in 2000, 2005 and 2010.

		2000	2005	2010
ESVs of the land use types	2000	—	Z = −2.201 (P = 0.028<0.05)	Z = −1.992 (P = 0.046<0.05)
	2005	Z = −2.201 (P = 0.028<0.05)	—	Z = −1.992 (P = 0.046<0.05)
	2010	Z = −1.992 (P = 0.046<0.05)	Z = −1.992 (P = 0.046<0.05)	—
ESVs of the land use types	2000	—	Z = −2.666 (0.008<0.01)	Z = −2.666 (0.008<0.01)
	2005	Z = −2.666 (0.008<0.01)	—	Z = −2.666 (0.008<0.01)
	2010	Z = −2.666 (0.008<0.01)	Z = −2.666 (0.008<0.01)	—

### Ecosystem Sensitivity Analysis

As is shown in Table 6, the CS in each case was less than 1, indicating that the estimated ESV was not affected by changes in the value coefficients. The CSs of grassland and forest land, with large areas and high value coefficients, were higher than those of the other land use types, with values of approximately 0.6 and 0.3, respectively. The areas of farmland and unused land were relatively large, but these types had low CSs and value coefficients. We can conclude from the estimated CSs that the calculated ESVs were responsible for the uncertainties in the value coefficients.

**Table 6:** Variation of the estimated total ecosystem service value (ESV) and the coefficient of sensitivity (CS) resulting from a 50% adjustment in the value coefficient in the national nature reserves in Ningxia in 2000, 2005, and 2010.

Land use type	Variation of ESV (%)			CS		
	2000	2005	2010	2000	2005	2010
Forest land	±16.33	±16.70	±16.85	0.33	0.33	0.34
Grassland	±28.82	±28.60	±28.21	0.58	0.57	0.56
Farmland	±3.05	±2.90	±2.99	0.06	0.06	0.06
Wetland	±0.03	±0.02	±0.01	0.00	0.00	0.00
Water body	±0.68	±0.73	±0.92	0.01	0.01	0.02
Unused land	±1.09	±1.06	±1.01	0.02	0.02	0.02
Construction land	±0.00	±0.00	±0.00	0.00	0.00	0.00

## Discussion

The recent enthusiasm for analyzing the concepts and methods for ES valuation appears to have been mostly initiated by the needs of conservationists to recommend broader and better-founded policies for protecting natural resources [43]. The responses of ecosystem quality to changes in land use and other factors are apparent in ESV variation. Economic analysis of ESs is an adequate framework for timely and effectively improving decisions involving various aspects of nature conservation and ecological restoration. Valuation may be a first step toward a “commodification” of nature and is not an end in itself but rather a conceptual and methodological framework for organizing information as a guide to making decisions and for managing ecological restoration and nature conservation.

Ecological restoration has been practiced in western China since the turn of the century. Through a wide range of comprehensive measures including policy, projects, technology, and capital, the trends of ecological degradation in the western region have been relieved. Ecological function is gradually being restored through the efforts of ecological restoration in weatern China. Variations in ESVs can reflect changes to the health of ecosystems and can thus evaluate the effects of these efforts at restoration. The evaluation of ESVs conducted in this study is a fast and effective way to assess the results of ecological restoration in western China. The increase of ESVs of the national nature reserves in Ningxia reflected the effects of ecological restoration to some extent. For example, the ESVs of forest land and water bodies increased with expanded areas and improved ecosystem quality (e.g. ecosystem productivity), and the ESVs of grassland, farmland, wetland, and unused land increased with improved ecosystem quality, even when the areas decreased. Water bodies, with a high value coefficient, had great potential for ESs. We thus recommend that more attention be paid to these land use types (e.g. water bodies) for ecological restoration and construction in the near future.

The method of deriving ESVs by multiplying the area of land use types and the value coefficients was proposed by Costanza et al. [20]. Their value coefficients were based on the average of global ecosystems, not tallied from the Chinese situation. Xie et al. [26] modified the value coefficients to apply to China. The accuracy and reliability of the evaluated results are mainly determined by the accuracy of the value coefficients. More accurate value coefficients are thus necessary. ESs have considerable spatial heterogeneity, so RS and GIS technology must be used to conduct ESs assessments to improve the reliability of ESVs at the regional scale. For our study, the protection of ecosystems in nature reserves confers advantages in ecosystem productivity and biodiversity, so parameter corrections to the value coefficients are needed for accurate estimation of the ESVs in nature reserves. With NPP and biodiversity parameter corrections on value coefficients, the ESV of biodiversity protection was the highest, and the ESV of food production was the lowest, in agreement with the actual situation of nature reserves in China.

Land use can be used as a proxy measure of ESs through matching land use types with equivalent biomasses and ESVs can be easily conducted based on land use data. Using the average value coefficients, however, may not be precise enough, because the land use classification was only applied to the first-level classification (e.g. forest land and grassland). The structural and functional differences of different ecosystems at the same level, e.g. the forest land type includes broad-leaved forests, coniferous forests, bush forests, etc., may lead to uncertain ESVs. Further studies, then, must apply the detailed land use classification and value coefficients to improve the understanding of the distributed characteristics of ESs.

Different methods of evaluation can provide different results. For example, both Li et al. [32] and Peng et al. [44] evaluated the ESV of Shenzhen for the same year, giving estimates of 2.9 billion and 126.5 billion Yuan, respectively. Absolute numbers of ESVs have less meaning, and the dynamics of ESVs are commonly indicating ecological problems. Despite the residual uncertainties due to the complex, dynamic, and nonlinear nature of ecosystems [45], [46], [47], accurate coefficients are often less critical for time-series than for cross-sectional analyses because value coefficients tend to affect estimates of directional change less than estimates of ecosystem values at specific points in time [32]. Our evaluation is thus valid for calculating ESVs over extended periods as a means of assessing the changes in ESVs in response to changes in land use.

## Conclusions

We assessed ESVs and their changes for national nature reserves in Ningxia from land use data obtained during 2000–2010. The areas of forest land, water bodies, and construction land increased, while the areas of grassland, farmland, wetland, and unused land decreased. Ecological restoration helped to increase the total ESV in the reserves during 2000–2010 from 182.3 million to 223.8 million US$, an increase of 22.7%. Grassland and forest land contributed approximately 90% of the total ESV. The ESVs of all ESs increased throughout the decade, especially biodiversity protection and soil formation and protection. We can thus conclude that ecological restoration in the national nature reserves in Ningxia during 2000–2010 had achieved good results.

By matching the land use types to equivalent biomes, ESV can be estimated by the land use data and the value coefficients using GIS and RS data. The value coefficients are the essential issue for the accuracy and reliability of ESV estimation. The coefficients of sensitivity indicated that the estimated ESVs were relatively rigid relative to the changes in the value coefficients. Our analysis of the variation in the ESVs in the national nature reserves in Ningxia will be able to serve as a reference for future analyses.
